# Supplementary material for: Bioinformatic Analysis and Machine Learning Methods in Neonatal Sepsis: Identification of Biomarkers and Immune Infiltration
Source: Biomedicines. 2023 Jun 28;11(7):1853. doi: 10.3390/biomedicines11071853 (PMC10377054; doi:10.3390/biomedicines11071853)
Supplement: Supplementary file 1 [file biomedicines-11-01853-s001.zip › biomedicines-2447955-supplementary.docx]

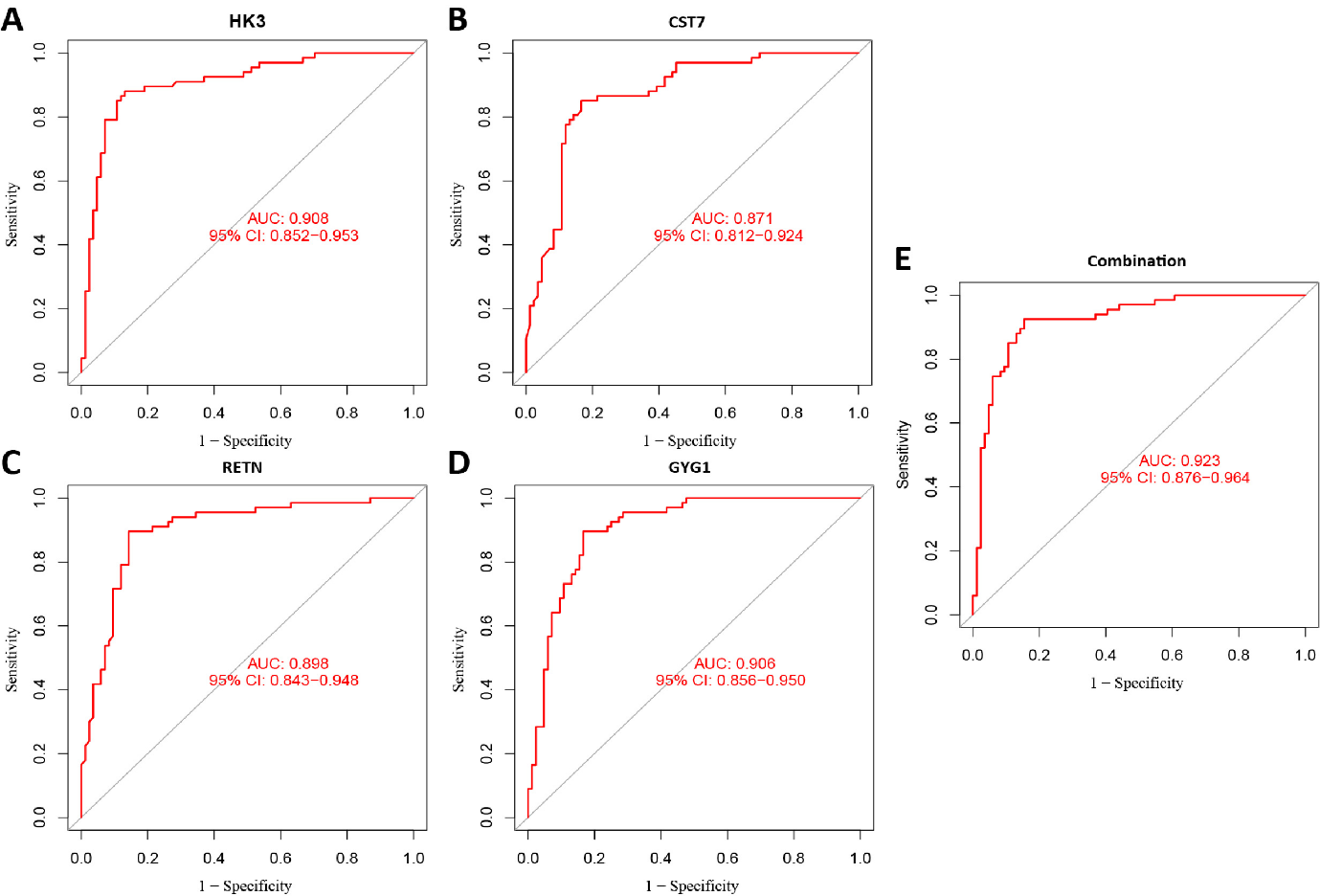


**Figure S1.** Diagnostic effectiveness of OFGs in testing dataset. (A-E) ROC curves for diagnostic effectiveness of HK3, CST7, RETN, GYG1 and combined in testing dataset.

**Table S1.** The thresholds setting and optimal feature genes in each ML method.

| **Machine learning** | **Thresholds Setting** | **Upregulated Optimal Feature Genes** | **Downregulated Optimal Feature Genes** |
| --- | --- | --- | --- |
| **LASSO** | The optimal penalty parameter was defined based on a 10-fold cross-validation minimum and used for each signature | HK3, GYG1, RETN, CST7, PROS1, HP, NKG7 | CLC |
| **SVM-RFE** | SVM-REF implements backward feature elimination and searches an optimal subset of features. | HK3, RETN, GYG1, CST7, PROS1, TDRD9, NLRC4 | CLC, LGALS2, LRRN3 |
| **Random Forest** | The parameter was 500 trees generated for each datapoint, and the meanDecreaseGini score >2. | HK3, CST7, RETN, GYG1, NLRC4, GPR84, SMPDL3A, ANKRD22 | LRRN3, |

**Table S2.** OFGs screened by machine learning algorithms in 10 trials.

| **Times Algorithms** | **LASSO** | **SVM-REF** | **RF** |
| --- | --- | --- | --- |
| 1 | GYG1 HK3 RETN CST7  PROS1 ANKRD22 TDRD9 METTL7B LRRN3 ALOX5AP OLAH PFKFB2 LCN2 LGALS2 CLC | GYG1 HK3 RETN CST7 GPR84 NLRC4 NSUN7 PROS1 IL1R2 ATP9A ZDHHC19 ANKRD22 LRRN3 TLR8 PSTPIP2 SORT1 DYSF ALOX5AP METTL7B VNN1 ALPL SAMSN1 PGD DHRS9 CLEC4D TDRD9 IL18R1 ANXA3 | GYG1 HK3 RETN CST7  NLRC4 IL1R2 GPR84 ANKRD22 ATP9A ZDHHC19 PROS1 LRRN3 |
| 2 | GYG1 HK3 RETN CST7 ZDHHC19 LRRN3  NSUN7 PROS1 LCN2 IFITM3 LGALS2 PRTN3 | GYG1 HK3 RETN CST7 LRRN3 ATP9A NSUN7 ZDHHC19 ALPL DYSF NLRC4 PGD PSTPIP2 PROS1 SORT1 IL1R2 GPR84 SMPDL3A ANKRD22 | GYG1 HK3 RETN CST7  ZDHHC19 LRRN3 DYSF NSUN7 NLRC4 IL1R2 ALPL |
| 3 | GYG1 HK3 RETN CST7 TDRD9 SMPDL3A SLCO4C1  PROS1 METTL7B IFITM3 PRTN3 | GYG1 HK3 RETN CST7 PROS1 NSUN7 NLRC4 ZDHHC19 PGD FPR1 TDRD9 IFITM3 FCER1G ALOX5AP PSTPIP2 SERPINB1 | GYG1 HK3 RETN CST7 NLRC4 FPR1 PSTPIP2 NSUN7 ZDHHC19 SERPINB1 PROS1 TDRD9 GPR84 |
| 4 | GYG1 HK3 RETN CST7 SMPDL3A NKG7PROS1 TDRD9 FCGR1B PRTN3 CLC | GYG1 HK3 RETN CST7 PROS1 SMPDL3A NLRC4 | GYG1 HK3 RETN CST7 NLRC4 GPR84 PROS1 SMPDL3A LRRN3 ALOX5AP PSTPIP2 |
| 5 | NSUN7 CD163 LRRN3 SMPDL3A IL18R1 IL1R2 PROS1 HP GIMAP7 SLCO4C1 S100A12 IL18RAP NKG7 MMP9 CLEC5A MS4A4A | LRRN3 NSUN7 HK3 PROS1 GIMAP7 RETN GYG1 IL1R2 NLRC4 ZDHHC19 PGD CST7 LGALS2 GPR84 CD3G PSTPIP2 IL18R1 NKG7 TDRD9 SLCO4C1 SORT1 SMPDL3A | LRRN3 HK3 NSUN7 NLRC4 PROS1 GYG1 PSTPIP2 GPR84 RETN GIMAP7 ZDHHC19 |
| 6 | GYG1 HK3 RETN CST7 NSUN7 HP PROS1 FCGR1A | GYG1 HK3 RETN CST7 NSUN7 NLRC4 PROS1 ZDHHC19 TDRD9 GPR84 SORT1 LRRN3 PSTPIP2 ANXA3 IL1R2 DYSF | GYG1 HK3 RETN CST7 NLRC4 NSUN7 GPR84 PROS1 ZDHHC19 IL1R2 ANKRD22 |
| 7 | GYG1 HK3 RETN CST7 NSUN7 TDRD9 DYSF PROS1 METTL7B SLCO4C1 FCER1G LCN2 | GYG1 HK3 RETN CST7 NSUN7 PGD NLRC4 PSTPIP2 IL1R2 PROS1 SMPDL3A ALPL DYSF GPR84 TLR8 SORT1 LRRN3 ZDHHC19 ALOX5AP TDRD9 IRAK3 ANXA3 MCTP2 ANKRD22 SLCO4C1 | GYG1 HK3 RETN CST7 NLRC4 NSUN7 GPR84 PROS1 PSTPIP2 |
| 8 | HK3 SMPDL3A PROS1  CST7 METTL7B HP FCGR1B FCER1G | HK3 PROS1 CST7 RETN NLRC4 GYG1 PSTPIP2 HP ALOX5AP NSUN7 OLAH TLR8 SMPDL3A PGD GPR84 ZDHHC19 LRRN3 FPR1 FCER1G TDRD9 KLRB1 | PROS1 FPR1 OLAH IL18R1 NSUN7 RETN |
| 9 | HK3 LRRN3 ZDHHC19 METTL7B SLCO4C1 PROS1 BASP1 S100A12 PFKFB2 NKG7 FCER1G CLEC5A LGALS2 CLC MPO | HK3 NLRC4 ZDHHC19 CST7 GYG1 RETN PGD PSTPIP2 | CST7 HK3 ZDHHC19 GYG1 SERPINB1 NSUN7 IL1R2 ITGAM PSTPIP2 SMPDL3A |
| 10 | GYG1 HK3 RETN CST7 GPR84 NLRC4 SORT1 PSTPIP2 ZDHHC19 ANKRD22 NSUN7 | GYG1 HK3 RETN CST7 GPR84 PGD PSTPIP2 PROS1 SORT1 NLRC4 ZDHHC19 ANKRD22  SERPINB1 NSUN7 ATP9A IL1R2 MCTP2 CEACAM1 ALOX5AP | GYG1 HK3 RETN CST7 NLRC4 NSUN7 GPR84 PSTPIP2 ZDHHC19  ANKRD22 SORT1 |

**Table S3.** The specific performance of the gene-models for four genes.

|  | **Training set** | | | | **Testing set** | | | |
| --- | --- | --- | --- | --- | --- | --- | --- | --- |
|  | **Accuracy** | **Precision** | **Recall** | **F1 value** | **Accuracy** | **Precision** | **Recall** | **F1 value** |
| HK3 | 0.85 | 0.79 | 0.90 | 0.84 | 0.84 | 0.79 | 0.88 | 0.83 |
| GYG1 | 0.85 | 0.79 | 0.90 | 0.84 | 0.87 | 0.82 | 0.91 | 0.86 |
| RETN | 0.85 | 0.79 | 0.90 | 0.84 | 0.82 | 0.76 | 0.85 | 0.81 |
| CST7 | 0.82 | 0.76 | 0.87 | 0.81 | 0.84 | 0.79 | 0.88 | 0.83 |
